# Periodicity of cerebral flow velocity during sleep and its association with white-matter hyperintensity volume

**DOI:** 10.1038/s41598-019-52029-4

**Published:** 2019-10-29

**Authors:** Woo-Jin Lee, Keun-Hwa Jung, Hyun-Min Park, Chul-Ho Sohn, Soon-Tae Lee, Kyung-Il Park, Kon Chu, Ki-Young Jung, Manho Kim, Sang Kun Lee, Jae-Kyu Roh

**Affiliations:** 1 Department of Neurology, Seoul National University Hospital, Seoul, South Korea; 2 Department of Radiology, Seoul National University Hospital, Seoul, South Korea; 3 Department of Neurology, Seoul National University Healthcare System Gangnam Center, Seoul, South Korea; 4 Protein Metabolism Research Center, Seoul National University College of Medicine, Seoul, South Korea; 5 Department of Neurology, The Armed Forces Capital Hospital, Sungnam, South Korea

**Keywords:** Neurodegeneration, Neurodegenerative diseases

## Abstract

Impaired sleep-related activation of the cerebral waste-clearance system might be related with the brain aging process. We hypothesized that cerebral blood-flow pattern changes during sleep might reflect the activation of the cerebral waste-clearance system and investigated its association with the cerebral white-matter hyperintensity (WMH) volume. Fifty healthy volunteers were prospectively recruited. In addition to the baseline transcranial Doppler parameters, the mean flow velocity (MFV) of the middle cerebral artery was monitored during waking and short-term non-REM sleep. Spectral density analysis was performed to analyze the periodic MFV variation patterns. For the aged subgroup (>50 years, n = 25), the WMH volumes in the total, subcortical, and periventricular regions were measured. The MFV periodic pattern during sleep was substantially augmented over that in the waking status. Spectral density analysis of MFV showed a noticeable peak in the very-low–frequency (VLF) band during sleep status (sleep/waking ratio 2.87 ± 2.71, *P* < 0.001). In linear regression analysis in the aged subgroup, the sleep/waking ratio of the VLF peak was inversely associated with total (*P* = 0.013) and subcortical (*P* = 0.020) WMH volumes. Sleep-related amplification of the cerebral flow-velocity periodicity might reflect the activation of cerebral waste clearance system during sleep, and be related to the pathogenesis of cerebral WMH.

## Introduction

Cerebral white matter hyperintensity (WMH) on brain magnetic resonance imaging (MRI) is commonly associated with the brain aging process and has been implicated in various complications including Alzheimer’s disease and other dementias^1,2^. Cerebral microvascular pathologies, including chronic subclinical hypoperfusion, arteriolosclerosis, and chronic inflammation have been widely accepted as the main pathomechanism of cerebral WMH^3–5^. Additionally, perivascular (glymphatic) system for cerebral waste clearance has been recently identified to have a major role in maintaining brain metabolic homeostasis^4,6^. As cerebral arteriolar wall motion during a cardiac cycle (pulsation) mainly drives the fluid exchange via the glymphatic system^7^, cerebral microvascular pathologies might be also closely related with the function of cerebral waste clearance system^5^.

Evidences from the recent studies suggest that sleep might be related with the cerebral waste clearance. Ooms *et al*. reported that one-night sleep deprivation is associated with increased concentration of β-amyloid 42 in cerebrospinal fluid (CSF), indicating that sleep might be implicated in the homeostasis of cerebral metabolic wastes^8^. Additionally, Xie *et al*. found that sleep dramatically facilitates the clearance of β-amyloid^9^. During sleep, the clearance rate of β-amyloid was two-fold faster than that during waking, and more than 70% of the injected β-amyloid was cleared out during the initial 30 min of sleep^9^, indicating that sleep might enhance the cerebral waste clearance system function and its integrity might be associated with the pathogenesis of cerebral WMH. Another study of Kiviniemi *et al*. using ultra-fast magnetic resonance encephalography reported that three distinct mechanisms regulates the glymphatic circulation, which are centrifugal cardiovascular pulsation that originates from basal peri-arterial spaces, respiration that regulates cortical perivenous spaces in a centripetal fashion, and the slow vasomotor oscillations in very-low and low frequencies (VLF and LF) which have distinct spatiotemporal patterns^10^. Taken together, there might be a sleep-associated cerebral hemodynamic regulation which involves in the facilitation of those mechanisms during sleep.

In this study, we hypothesized that changes in the cerebral blood-flow parameters in the early sleep phase might reflect the activation of the waste-clearance system during sleep and may therefore contribute to the pathogenesis of cerebral WMH. In this regard, we investigated the changes in blood-flow parameters during short-term non-rapid eye movement (non-REM) sleep in young and aged populations with no reported neurological diseases using transcranial Doppler (TCD) monitoring, and its association with WMH volume on brain MRI.

## Results

Fifty participants, including 37 males (74.0%) and 13 females (26.0%), with a mean age of 47.3 ± 12.6 years (range, 24–69 years) were included. The average duration of sleep during the three consecutive nights prior to the evaluation was 6.4 ± 1.2 h (range, 4.0–10.5 h). In the sleep monitoring, the mean duration of waking status was 299.4 ± 95.6 s (range, 134–729 s), and the mean duration of non-REM sleep status was 877.1 ± 321.3 s (range, 603–1683 s). Compared to the group aged ≤50 years, the group aged >50 years showed a higher frequency of hypertension and diabetes mellitus, a higher risk of obstructive sleep apnea (OSA), lower mean flow velocity (MFV), higher Epworth sleepiness scale (ESS) scores, and higher pulsatility index (PI) values during both waking and sleep statuses (Table 1).

**Table 1:** Clinical, sleep questionnaire, transcranial Doppler, and white matter hyperintensity profiles of the study population.

	Age ≤ 50 years N = 25	Age > 50 years N = 25	*P*
**Clinical profiles**			
Age (year)	36.1 ± 5.8	58.5 ± 5.5	<0.001**
Male sex	16 (64.0)	21 (84.0)	0.066
Body mass index (kg/m^2^)	23.1 ± 3.6	24.3 ± 2.9	0.192
Hypertension	2 (8.0)	12 (48.0)	0.001^**^
Diabetes mellitus	0 (0.0)	5 (20.0)	0.022^*^
Hyperlipidemia	0 (0.0)	2 (8.0)	0.162
Use of ACEi/ARB	1 (4.0)	9 (36.0)	0.005^**^
Use of calcium channel blocker	0 (0.0)	4 (16.0)	0.043^*^
Smoking in past 5 years	8 (32.0)	13 (52.0)	0.098
Systolic blood pressure (mmHg)	125.6 ± 13.0	129.5 ± 15.2	0.324
Diastolic blood pressure (mmHg)	74.5 ± 7.8	77.1 ± 9.0	0.275
Mean blood pressure (mmHg)	108.6 ± 9.9	112.1 ± 12.4	0.270
Heart rate (no./min)	72.0 ± 8.3	74.8 ± 10.7	0.294
**Sleep questionnaire profiles**			
Average sleep duration (hours)^†^	6.4 ± 1.1	6.4 ± 1.4	0.885
ESS score (0−24)	3 (1.75–4)	5 (4–10)	0.007^**^
ESS score > 10	2 (7.7)	5 (20.8)	0.197
High risk of OSA^‡^	9 (36.0)	16 (64.0)	0.023^*^
**Waking TCD parameters**			
Evaluation time (sec)	300.2 ± 67.8	298.6 ± 70.6	0.956
MFV (cm/sec)	68.2 ± 10.0	60.2 ± 11.8	0.012*
PI	0.69 ± 0.11	0.78 ± 0.13	0.005**
MFV variation ([cm/sec]^2^)	10.7 ± 14.4	7.8 ± 5.2	0.360
**Spectrum density analysis**			
ULF relative power (%)	7.2 ± 4.4	7.1 ± 4.4	0.937
VLF relative power (%)	59.4 ± 16.6	65.0 ± 16.0	0.235
LF relative power (%)	32.8 ± 18.0	26.6 ± 16.7	0.215
HF relative power (%)	0.6 ± 0.7	1.3 ± 2.3	0.139
Power of VLF peak frequency during sleep ([cm/sec]^2^)	3246.4 ± 6192.9	2118.8 ± 1812.0	0.395
**Sleep TCD parameters**			
Evaluation time (sec)	910.7 ± 300.6	840.8 ± 145.1	0.448
MFV (cm/sec)	69.7 ± 8.9	61.9 ± 11.2	0.009**
PI	0.6 ± 0.1	0.7 ± 0.1	<0.001**
MFV variation ([cm/sec]^2^)	30.2 ± 12.9	27.2 ± 15.0	0.443
**Spectrum density analysis**			
ULF relative power (%)	11.6 ± 4.1	9.6 ± 8.6	0.311
VLF relative power (%)	77.6 ± 6.2	81.8 ± 9.3	0.068
LF relative power (%)	10.8 ± 3.8	8.6 ± 5.4	0.104
HF relative power (%)	0.0 ± 0.1	0.0 ± 0.1	0.680
Peak frequency of VLF band (Hz)	0.013 ± 0.005	0.014 ± 0.005	0.305
Power of VLF peak ([cm/sec]^2^)	15433.8 ± 9759.4	18881.2 ± 14730.4	0.323
**Sleep/Waking Ratios**			
Mean MFV	1.0 ± 0.1	1.0 ± 0.0	0.648
Mean PI	0.8 ± 0.2	0.9 ± 0.	0.087
MFV variation	5.3 ± 5.8	5.1 ± 6.2	0.909
VLF relative power	1.2 ± 0.4	1.4 ± 0.7	0.274
LF relative power	0.4 ± 0.3	0.6 ± 0.5	0.111
HF relative power	0.0 ± 0.1	0.1 ± 0.4	0.194
Relative power of VLF peak	3.1 ± 3.7	2.7 ± 1.8	0.617
**WMH volume parameters**			
Total WMH (mL)	—	2.8 ± 2.3	—
Subcortical WMH (mL)	—	1.3 ± 1.1	—
Periventricular WMH (mL)	—	1.6 ± 1.7	—

Data are reported as a number (percentage), mean ± standard deviation, or median (interquartile range, IQR). ACEi/ARB, angiotensin converting enzyme inhibitor/angiotensin receptor blocker, ESS, Epworth sleepiness scale, OSA, obstructive sleep apnea, MFV, mean flow velocity, PI, pulsatility index, ULF, ultra-low frequency band, VLF, very low frequency band, LF, low frequency band, HF, high frequency band, and WMH white matter hyperintensity. ^†^Average sleep duration of the last 3 nights prior to the evaluation, ^‡^Determined per the criteria of the Berlin questionnaire, ^*^P < 0.05, ^**^P < 0.01.

**Figure 1:**
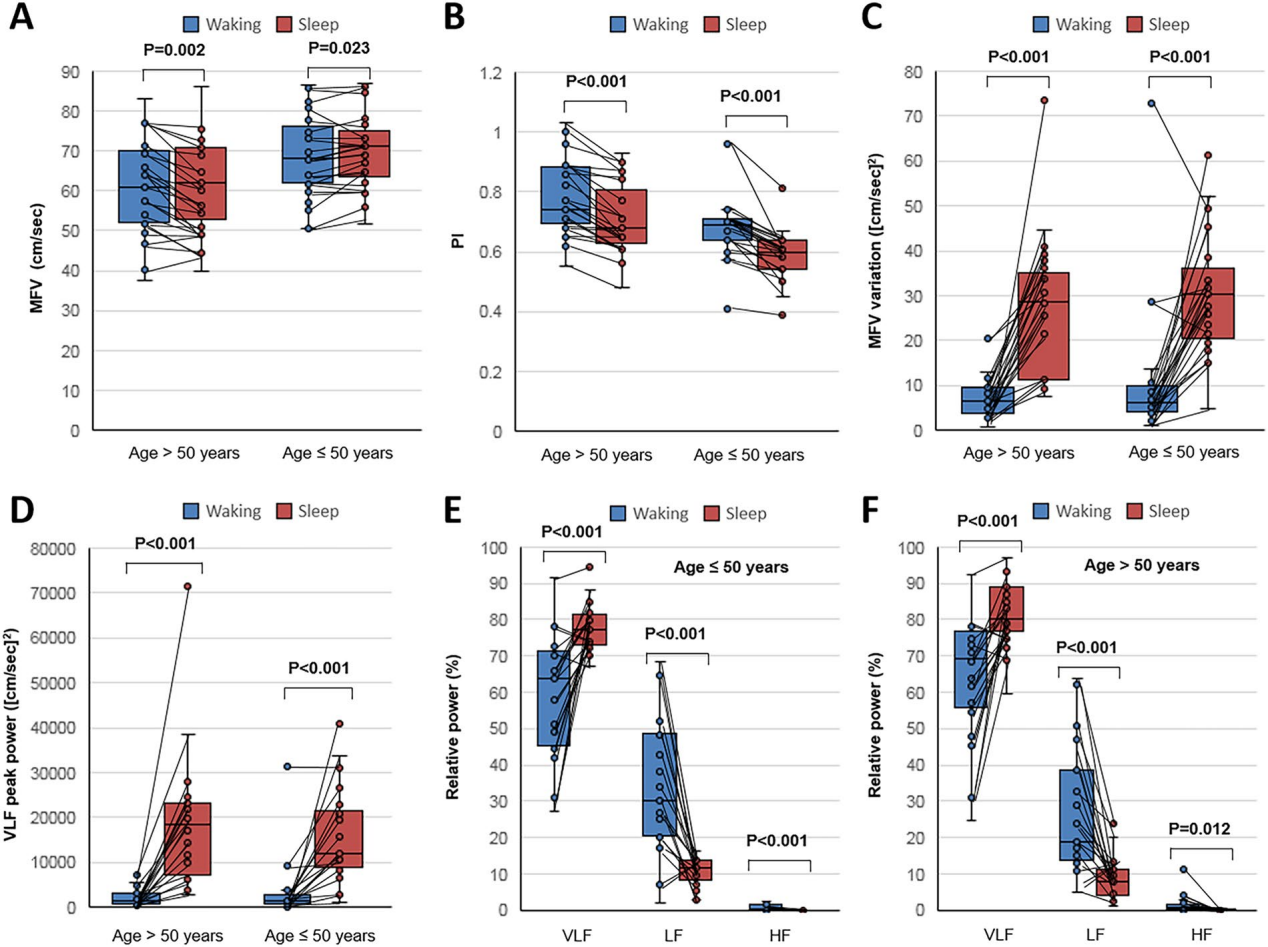
Comparison of flow velocity parameters between the waking and sleep stages. Subjects were divided into two subgroups according to age (≤50 and >50 years). In each subgroup, the mean flow velocity (MFV) increased (panel A), the pulsatility index (PI) decreased (panel B), the variance of MFV increased (panel C), and the absolute power of the peak in the very-low–frequency (VLF) band (panel D) and the relative power of the VLF band in the spectral density analysis (panels E and F) significantly increased during the sleep status, compared to the waking status. The sleep/waking ratios of these parameters were not significantly different between the age subgroups. Horizontal lines above the bars denote standard errors. Abbreviations: ULF, ultra-low frequency; LF, low frequency; HF, high frequency.

**Figure 2:**
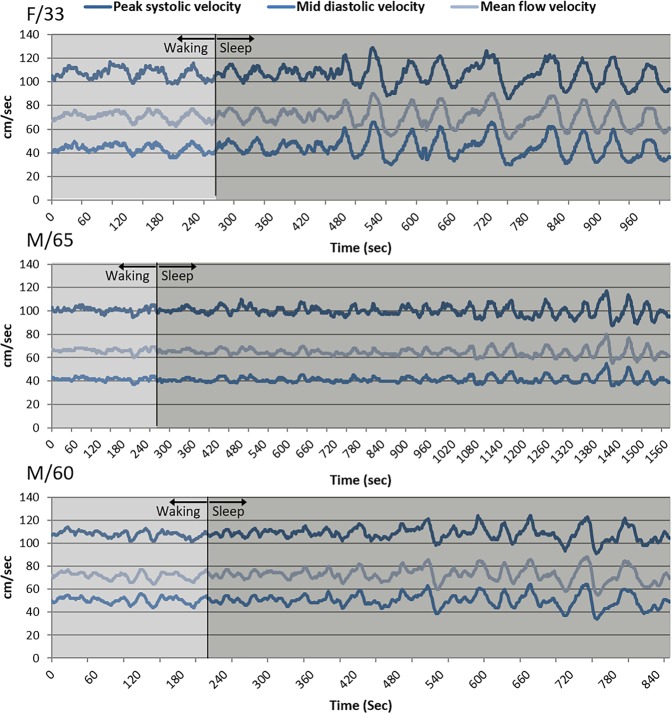
Changes in the mean flow velocity in sleep monitoring: Representative cases. Transcranial Doppler monitoring during short-term non-REM sleep in a 33-year-old woman (upper panel), a 65-year-old man (middle panel), and a 60-year-old man (lower panel) showed a marked amplification of the periodic patterns in the peak systolic velocity, mid-diastolic velocity, and mean flow velocity graphs with a period of approximately 50 s, which appeared within a few minutes after the onset of sleep.

**Figure 3:**
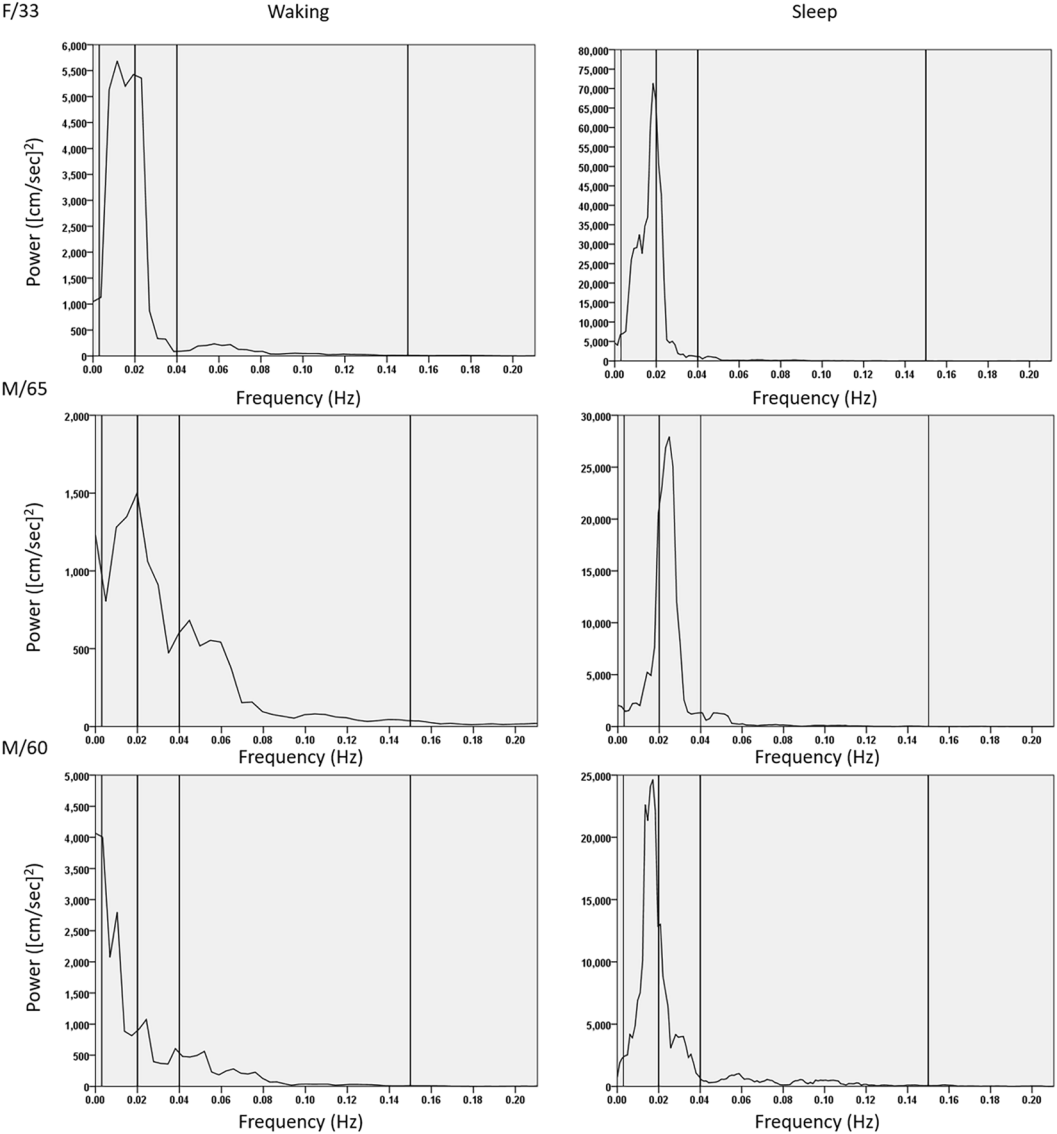
Spectral density analysis of the mean flow velocity: Representative cases. To analyze the periodic component of the mean flow velocity (MFV) variation, a fast Fourier transformation was applied and the graphs of spectral density according to the frequency domain were drawn for a 33-year-old woman (upper panel), a 65-year-old man (middle panel), and a 60-year-old man (lower panel). As the result, a peak was observed in the very-low–frequency (VLF, 0.003–0.04 Hz) band during the sleep status (right panels), which was not prominent during the waking status (left panels).

Compared to the findings in the waking status, the MFV modestly increased and PI decreased during the sleep status in both age groups (Fig. 1A,B). However, a substantial increment in MFV variation was observed in the sleep status (Fig. 1C). When graphing the MFV change over time, a periodic pattern in MFV variation with a period of approximately 50 s appeared within a few minutes after the onset of sleep in most patients (Fig. 2). When a spectral density analysis was applied to the MFV-time data, a peak in the VLF band during the sleep status appeared in the majority of subjects, which was not evident during the waking status (Fig. 3). Accordingly, in both age subgroups, the absolute power of this peak in the VLF band and the relative power of the VLF band significantly increased during the sleep status compared to the waking status (Fig. 1D–F and Table 1). The mean sleep/waking ratio of the VLF peak was 2.9 ± 2.7, indicating that the periodic component of the MFV relevant to the VLF peak frequency increased by approximately three times during non-REM sleep.

Correlation analyses revealed that the sleep/waking ratio of the VLF peak was highly correlated with the sleep/waking ratio of MFV variation (*r* = 0.724, *P* < 0.001) and showed inverse correlation with the average sleep duration (*r* = −0.318, *P* = 0.041), whereas other variables such as age, blood pressure parameters, TCD parameters, and sleep monitoring parameters did not show any significant correlation (Supplemental Table S1). Among categorical parameters, none of the sex, vascular risk factors, medications, ESS score of >10, or a high risk of OSA were significantly associated with higher sleep/waking ratio of MFV variation (Supplemental Table S2). The sleep/waking ratios of MFV, PI, MFV variation, relative power of each frequency band, and the relative power of the peak in the VLF band were comparable between the two age groups (Table 1).

In the WMH volume analysis in the subgroup aged >50 years, the mean total WMH volume was 2.8 ± 2.3 mL, subcortical WMH volume was 1.3 ± 1.1 mL, and periventricular WMH volume was 1.6 ± 1.7 mL. Correlation analyses revealed that total WMH volume was associated with increasing age (*r* = 0.453, *P* = 0.026) and lower sleep/waking ratio of MFV variation (*r* = −0.434, *P* = 0.034); the subcortical WMH volume was associated with lower sleep/waking ratio of MFV variation (*r* = −0.499, *P* = 0.013) and the lower sleep/waking ratio of the VLF peak (*r* = −0.406, *P* = 0.049); and the periventricular WMH volume was associated with increasing age (*r* = 0.463, *P* = 0.023) (Table 2). Among categorical parameters, none of the sex, vascular risk factors, medications, or a high risk of OSA were significantly associated with higher values for the three WMH volume categories (Table 3). Due to high co-linearity, the sleep/waking ratios of MFV variation were discarded and only the sleep/waking ratios of the VLF peak were included in the multivariate analysis.

**Table 2:** Correlation coefficients of white matter hyperintensity volume with continuous variables.

WMH volume (mL)	Total	*P*	Subcortical	*P*	Periventricular	*P*
Age (year)	0.453	0.026^*^	0.075	0.726	0.463	0.023^*^
Body mass index (kg/m^2^)	−0.137	0.524	−0.116	0.591	−0.114	0.594
Systolic blood pressure (mmHg)	0.029	0.894	0.093	0.666	−0.221	0.300
Diastolic blood pressure (mmHg)	−0.020	0.927	0.015	0.944	−0.173	0.418
Mean blood pressure (mmHg)	0.019	0.931	0.061	0.776	−0.223	0.296
Epworth sleepiness score	−0.085	0.693	−0.020	0.926	0.001	0.997
Waking MFV	−0.161	0.453	0.117	0.588	−0.067	0.757
Waking PI	0.277	0.191	0.036	0.867	0.312	0.138
MFV (Sleep/Waking Ratio)	0.035	0.872	0.128	0.512	−0.020	0.928
PI (Sleep/Waking Ratio)	−0.088	0.682	−0.185	0.386	0.130	0.546
MFV variation (Sleep/Waking Ratio)	−0.434	0.034^*^	−0.499	0.013^*^	0.083	0.698
VLF relative power (Sleep/Waking Ratio)	−0.037	0.865	−0.219	0.303	0.031	0.886
LF relative power (Sleep/Waking Ratio)	−0.242	0.255	0.241	0.256	0.178	0.407
HF relative power (Sleep/Waking Ratio)	0.237	0.265	0.006	0.977	−0.076	0.725
Sleep/waking ratio of the VLF peak	−0.349	0.094	−0.406	0.049^*^	−0.111	0.613

MFV, mean flow velocity, PI, pulsatility index, VLF, very low frequency bend, LF, low frequency bend, HF, high frequency bend, and WMH white matter hyperintensity. ^*^P < 0.05, ^**^P < 0.01.

**Table 3:** Univariate analyses for factors associated with white matter hyperintensity severity.

	Total WMH (mL)			Subcortical WMH (mL)			Periventricular WMH (mL)		
	No	Yes	*P*	No	Yes	*P*	No	Yes	*P*
Male sex	2.92 ± 2.18	3.95 ± 2.30	0.472	1.45 ± 1.18	1.93 ± 1.11	0.486	1.47 ± 1.02	2.02 ± 1.75	0.605
Hypertension	3.74 ± 2.01	3.91 ± 2.60	0.862	1.83 ± 1.01	1.91 ± 1.23	0.862	1.91 ± 1.72	1.99 ± 1.67	0.903
Use of ACEi/ARB	2.39 ± 1.94	3.54 ± 2.70	0.237	1.06 ± 0.98	1.63 ± 1.26	0.224	1.33 ± 1.58	1.91 ± 1.84	0.422
Use of calcium channel blocker	2.77 ± 2.03	3.06 ± 3.64	0.821	1.27 ± 1.10	1.27 ± 1.29	0.996	1.50 ± 1.48	1.80 ± 2.71	0.845
Diabetes mellitus	3.72 ± 2.25	4.20 ± 2.55	0.684	1.76 ± 1.01	2.30 ± 1.44	0.340	1.96 ± 1.80	1.90 ± 1.12	0.944
Hyperlipidemia	3.84 ± 2.36	3.68 ± 0.96	0.928	1.85 ± 1.14	2.17 ± 0.43	0.701	1.99 ± 1.73	1.51 ± 0.52	0.705
Smoking in past 5 years	3.21 ± 2.31	4.33 ± 2.18	0.233	1.52 ± 0.89	2.17 ± 1.20	0.150	1.70 ± 1.61	2.17 ± 1.74	0.503
ESS score > 10	3.48 ± 2.06	5.13 ± 2.81	0.194	1.76 ± 1.05	2.36 ± 1.30	0.335	1.72 ± 1.51	2.82 ± 2.10	0.152
High risk of OSA	4.33 ± 2.56	3.57 ± 2.16	0.454	1.91 ± 1.27	1.85 ± 1.05	0.907	2.42 ± 2.08	1.72 ± 1.43	0.344

Data are reported as mean ± standard deviation. ACEi/ARB, angiotensin converting enzyme inhibitor/angiotensin receptor blocker, ESS, Epworth sleepiness scale, and OSA, obstructive sleep apnea, ^*^P < 0.05.

In multivariate linear regression analyses, the sleep/waking ratio of the VLF peak was associated with smaller total (B coefficient = −0.517; 95% confidence interval [CI] −0.915–−0.120; *P* = 0.013) and subcortical WMH volumes (B = −0.630; 95% CI −1.150–−0.109; *P* = 0.020), but not associated with periventricular WMH volume. Age was significantly associated with larger total (B = 0.092; 95% CI 0.033–0.151; *P* = 0.004) and higher periventricular WMH volume (B = 0.095; 95% CI 0.025–0.164; *P* = 0.010), but not with subcortical WMH volume (Table 4). In the scatterplot of the standardized predicted values and the standardized residuals, a random and even distribution of the standardized residuals around the zero line was observed. VIF values for each variable were <1.5.

**Table 4:** Linear regression analyses for factors associated with white matter hyperintensity severity.

Total WMH^†a^	B (95% CI)	β	*P*
Constant variable	1.289 (−2.267–4.844)		0.458
Age	0.092 (0.033–0.151)	0.543	0.004^*^
Sleep/waking ratio of the VLF peak^†^	−0.517 (−0.915–−0.120)	−0.455	0.013^*^
**Subcortical WMH** ^†b^			
Constant variable	2.524 (−2.131–7.178)		0.271
Age	0.049 (−0.028–0.127)	0.244	0.198
Sleep/waking ratio of the VLF peak^†^	−0.630 (−1.150–−0.109)	−0.465	0.020^*^
**Periventricular WMH** ^†c^			
Constant variable	−0.335 (−4.807–4.137)		0.877
Age	0.106 (0.035–0.177)	0.571	0.006^*^
Sleep/waking ratio of the VLF peak^†^	−0.134 (−0.346–0.077)	−0.243	0.201
Waking PI	1.701 (−1.338–4.740)	0.210	0.257

^a^R^2^ = 0.497 and *P* = 0.003, ^b^R^2^ = 0.391 and *P* = 0.017, and ^c^R^2^ = 0.369 and *P* = 0.024 for the linear regression equations.

^†^The variables were log transformed to obtain a normal distribution. B, unstandardized coefficient and β, standardized coefficient. ^*^P < 0.05, ^**^P < 0.01.

## Discussion

In the present study, we observed a marked amplification of the periodic pattern of middle cerebral artery (MCA) MFV variation with a VLF occurring shortly after the onset of sleep, which resulted in a high peak power density in the VLF band in spectral density analysis during non-REM sleep. Notably, the sleep/waking ratio of the VLF peak was associated with the total and subcortical WMH volumes, after adjusting age and sex.

Numerous evidences have indicated that sleep disturbances such as decreased sleep quality^11^, short sleep duration^12^, and sleep-disordered breathing^13^ are highly associated with cognitive impairment in aged population. Increased concentration of β-amyloid in the brain after an inadequate sleep has been recognized^8^, and it is proposed that sleep deprivation increases the production of β-amyloid^14^. Association between sleep apnea and increased WMH volume has been reported and its pathophysiologic basis was presumed to be chronic inflammation and activation of stress hormones in the brain^15^. Regarding OSA, a negative correlation between slow-wave EEG activities in polysomnography and CSF β-amyloid levels in the following morning was found in healthy patients but not in OSA patients, indicating that OSA might interfere with the clearance of cerebral metabolites^16^.

The pathophysiologic link between the sleep-related amplification of MFV periodicity and the volume of WMH might be postulated as follows: First, sleep-related amplification of MFV periodicity might reflect the cerebral microvacsular compliance, of which reduction is the main mechanism underlying the progression of WMH. Since sleep regulates the autonomic influence toward a reduction in the sympathetic tone, sleep would be able to increase arterial compliance and amplify the variabilities in cerebral blood flow. However, the capacity of amplifying the blood flow variability might be largely depend on the structural properties of cerebral microvessels^6,17^. Second, intracranial pressure (ICP) also oscillates synchronously with the MFV oscillation, known as the B-wave of ICP^18–20^. Therefore, amplification of the MFV oscillation might induce a periodic fluctuation in the hydrostatic pressure of the CSF^18^, facilitate the convection of CSF to ISF, and consequently enhance cerebral waste clearance during sleep^4–6,9^. Third, since the regulation of B-waves is independent from the pCO_2_ level, blood pressure, respiration, central venous or airway pressure^21^, the MFV periodicity might represent a distinct motive of glymphatic system regulation and its sleep-related amplification might reflect the enhanced glymphatic function during sleep. A recent study using ultra-fast magnetic resonance encephalography revealed a vasomotor oscillation in VLF band that have distinct spatiotemporal patterns from those generated from cardiac or respiratory activities. In that study, the authors argued that this VLF oscillation might represent a distinct motive of regulating glyamphtic system function^10^. Taken together, sleep-induced amplification of MFV oscillation might be a distinctly regulated physiologic process that aims to enhance the efficacy of cerebral waste clearance and reflect the cerebral microvascular compliance.

The sleep/waking ratio of the VLF peak power was associated with the subcortical and total WMH volumes, but not with the periventricular WMH volume. This sight-specificity might be explained by that the periventricular white matter is mainly supplied by the penetrating arterioles from the MCA whereas the subcortical region is mainly supplied by the leptomeningeal branches^17,22^. Compared to the leptomeningeal branches, the MCA-penetrating arterioles have higher resistance and the CSF-ISF exchange via the paravascular space of the MCA-penetrating arterioles might be determined by intrinsic arteriolar compliance rather than dynamic changes in cerebral blood flow^17^. However, arteriolar compliance marker such as MCA PI was not significantly associated with the WMH volume. This might be due to the relatively low mean age of the current study population, as PI reflects the long-term process of vascular aging^23^.

The present study has some limitations to be addressed. First, due to that study was performed during daytime and the short duration of monitoring, the findings of this cannot be directly applied to the relationship between the cerebral WMH and the hemodynamic changes during nocturnal long-term sleep. In this study, the examiner had to hold the probe to maintain a fixed angle and insonation depth that best evaluates the MCA flow velocities throughout the monitoring. In this regard, it was impossible to perform long-term sleep monitoring. Advanced analyses such as correlating the VLF periodicity changes to EEG spectral plots or separately evaluating the VLF periodicity in different sleep stages might help more directly elucidate the mechanism and the physiologic role of VLF oscillation of cerebral blood flow during sleep. However, those evaluations were unavailable in this study setting, as they required long-term monitoring data. Second, some factors such as presence of OSA, use of ACEi/ARB or calcium-channel blockers, or whether a participant is better in taking daytime sleep, might possibly affect the VLF periodicity changes during short-term sleep, but their effect were not fully evaluated in this study. Third, due to the small sample size, only a few variables were included in the multivariate analyses. Fourth, due to the cross-sectional design, this study did not establish a causative relationship between a diminished sleep/waking ratio of the VLF peak power and the progression of WMH. Fifth, this study did not include patients with higher WMH volumes. Future studies with larger study populations, including a healthy geriatric population as well as dementia patients, follow-up MRI analyses, and whole-night monitoring of MFV are warranted to clarify the clinical significance of sleep-related amplification of MFV oscillation in the pathomechanism underlying cerebral WMH.

In conclusion, sleep-related amplification of the cerebral flow oscillation, measured as the sleep/waking ratio of VLF peak power in the spectral analysis of the flow velocity, was associated with total and subcortical WMH volumes.

## Materials and Methods

### Study subjects

Fifty volunteers were prospectively recruited from the local community. Participants were cognitively normal, had no history of any neurological disease, had normal sleep quality defined as a Pittsburgh Sleep Quality Index of 4 or less, had no significant intracranial arterial stenosis evaluated by TCD or MR angiography nor a preexisting ischemic/hemorrhagic lesions in MRI^24,25^, and had no medications that regulate alertness, affect sleep structure, or alter the vascular tone. However, patients who were taking angiotensin converting enzyme inhibitors/angiotensin receptor blockers (ACEis/ARBs) or calcium-channel blockers to treat hypertension were included in this study. Participants were categorized according to their ages into those aged ≤50 years (young subgroup, n = 25) and those aged >50 years (aged subgroup, n = 25)^17,26^. This study was approved by the institutional review board of Seoul National University Hospital (SNUH). All methods were performed in accordance with the institutional review board of SNUH regulations and STROBE guidelines for observational studies. Written informed consent was obtained from all participants.

### Acquisition of clinical data

Clinical profiles that included demographic data and information regarding body mass index (BMI, kg/m^2^) and the presence of hypertension, diabetes mellitus, hyperlipidemia, and a smoking history in the past five years were obtained^17,26^. Systolic, diastolic, and mean blood pressure (SBP, DBP, and MBP, all mmHg), and heart rate (HR,/min) were obtained using an electronic manometer after more than 5 min of rest in the sitting position.

### Sleep questionnaire

Subjects were encouraged to sleep regularly for more than three consecutive nights prior to the day of TCD monitoring. Before the evaluation, data for the following sleep questionnaires were collected: Berlin questionnaire (BQ, for screening a high risk of obstructive sleep apnea)^27^, ESS (a rating scale of 0 to 24 evaluating the likelihood of dozing or falling asleep during routine daily activities. ESS scores of >10 indicate significant sleepiness)^28^, and self-reported latency (min) and average sleep duration (h) in the latest three days.

### Transcranial doppler evaluation

Intracranial arteries were sonographically evaluated using a 2-MHz pulsed-wave and range-gated TCD probe (ST3 Digital PMD 150; Spencer Technologies; Redmond, WA, USA) with a transmission power level of 100 mW/cm^2^, pulse repetition frequency of 8000 Hz, filter frequency of 200 Hz, gain of 4 decibels, and range of 200 decibels. The evaluations were initiated between 2–4 PM. Throughout the evaluation, the laboratory room was maintained in a light-off noiseless state. The TCD evaluation protocol was standardized for every patient and was conducted by a TCD sonographer with 15 years of experience.

Before the monitoring, the flow in the MCA, the anterior cerebral artery, and the internal carotid artery (ICA) was evaluated to exclude stenosis of the anterior cerebral arteries. Then, TCD parameters, including peak systolic, minimal diastolic, and mean flow velocities (PSV/MDV/MFV, cm/s) and the pulsatility index (PI) were obtained along the MCA with insonation depths of 64, 60, 56, 50, and 46 mm^25,29^.

### Sleep monitoring

For sleep monitoring, two TCD probes were positioned on both temporal windows using a marc 600 headframe (Spencer Technologies), and the positions of the probes were adjusted until a maximal signal with an insonation depth of 56 mm was obtained. The side of a higher flow velocity was selected for monitoring and PSV, MDV, and MFV were recorded every second throughout the monitoring^30^. To assess the sleep-waking state, electroencephalography (EEG) using six electrodes (F3, F4, C3, C4, O1, and O2) and electrooculogrpahy (EOG) were monitored during the examination^28,30^. The sleep onset was defined according to the American Academy of Sleep Medicine scoring manual^31^. When the patients re-entered to wake stage after the onset of sleep, the monitoring was terminated. The short duration of monitoring is due to the technical difficulty that the examiner had to hold the probe to maintain a fixed angle and insonation depth that best evaluates the MCA flow velocities throughout the study. In this regard, it was impossible to perform TCD monitoring during long-term sleep. As REM sleep stage is related with cerebral blood flow parameters profiles comparable to those of waking status^30,32^, only the initial non-REM sleep period from the sleep onset were included in the analyses.

### Analysis of blood-flow parameters

For both sleep and waking statuses, the mean values of MFV and PI and the variations in MFV were measured. To evaluate the periodic component of MFV variation, a fast Fourier transformation was applied to the MFV data for each status^29^. A spectrum density analysis was then performed to express the power ([cm/s]^2^) as a function of frequency (Hz). The frequency domain was divided into four frequency bands: the ultra-low–frequency (ULF, <0.0003 Hz), very-low–frequency (VLF, 0.003–0.04 Hz), low-frequency (LF, 0.04–0.15 Hz), and high-frequency (HF, >0.15 Hz)^33,34^. The relative power of VLF, LF, and HF bands (%) was calculated by numerical integration of powers in the domain of each frequency band, divided by the numerical integration of powers in the total frequency domain. Since a long recording time (>12 h) is a prerequisite for reliable assessments of the ULF domain parameters, the ULF data was discarded^34^.

To evaluate sleep-related changes in these flow parameters, the sleep/waking ratios of the MFV, PI, MFV variations, and the relative power of each frequency band were calculated. Since a noticeable peak appeared at the VLF band during the sleep status, we also evaluated the sleep/waking ratio of the normalized power of the VLF peak. First, in the spectral density graph of the sleep status, the normalized absolute power of the VLF peak was calculated by dividing the absolute power of the VLF peak by the numerical integration of power throughout the total frequency domain. Second, in the spectral density graph of the waking status, the normalized power of the VLF peak frequency of the waking status was calculated in the same way. Third, the sleep/waking ratio of the VLF peak was calculated by the normalized power of the VLF peak in the sleep status divided by that in the waking status.

### Analysis of WMH volume

In the group of patients aged >50 years, MRI was performed using a 1.5-T unit (Ingenia; Philips, Best, Netherlands) according to protocols that included T1/T2-weighted imaging, the fluid-attenuated inversion recovery (FLAIR) and gradient echo (GRE) sequences, and time-of-flight magnetic resonance angiography (MRA). FLAIR sequences were obtained with the following parameters: slice thickness/gap of 4.0/0.0 mm, 24–27 slices covering the entire brain, repetition time/echo time (TR/TE) = 9000–9900/97–163 ms, a field-of-view (FOV) = 240 × 240 mm, and matrix = 220 × 220. FLAIR and GRE images were reviewed to exclude preexisting ischemic or hemorrhagic lesions, and MRA was performed to exclude significant stenosis in cerebral arteries.

For volumetric analysis of WMH, FLAIR sequences were registered into an offline workstation. Using a semi-automated freeware NeuRoi (Nottingham university, Nottingham, UK)^17,26^, the WMH lesion was identified and classified as periventricular or deep subcortical and its boundaries were drawn and volume was calculated by a neurologist (WJL, with 7 years of experience and blinded to other data) according to previously described protocols of which the reproducibility was established^17,26^.

### Statistical analysis

For all statistical analyses, SPSS 21.0 (SPSS Inc., Chicago, IL, USA) was used. Data are reported as numbers (percentage), mean ± standard deviations, or as medians (interquartile range, IQR). Pearson coefficients or Spearman’s test were used to measure correlations between continuous or ordinal variables and WMH volume. Paired *t* test was performed to compare the blood flow parameters between sleep and waking status. Student’s *t* tests or Mann–Whitney *U* tests were used to compare the sleep/waking ratio of the VLF peak power and the WMH volume among dichotomized subgroups. Variables with *P* values < 0.15 in univariate analyses were included in a multivariate linear regression analysis using an enter method. Age and sex were adjusted in the multivariate regression analysis regardless of the significance of the association in univariate analyses. In the multivariate analysis, WMH volume parameters and parameters from the spectral density analysis were log-transformed to obtain a normal distribution. The variance inflation factor (VIF) was measured to verify the multi-collinearity between variables. A scatterplot of the standardized predicted values and the standardized residuals was made to check the assumption for linearity of the regression model. For all analyses, *P* values < 0.05 were considered to be statistically significant.

## Supplementary information

Online Supplements

## Data Availability

The datasets generated during and/or analysed during the current study are available from the corresponding author on request.
