# Supplementary material for: Periodicity of cerebral flow velocity during sleep and its association with white-matter hyperintensity volume
Source: Sci Rep. 2019 Oct 29;9:15510. doi: 10.1038/s41598-019-52029-4 (PMC6820785; doi:10.1038/s41598-019-52029-4)
Supplement: Supplementary file 1 — Online Supplements [file 41598_2019_52029_MOESM1_ESM.docx]

Online Supplements

**Periodicity of cerebral flow velocity during sleep and its association with white-matter hyperintensity volume**

Woo-Jin Lee, MD^a^; Keun-Hwa Jung, MD, PhD^a^; Hyun-Min Park, RVT, MS^a^; Chul-Ho Sohn, MD, PhD^b^; Soon-Tae Lee, MD, PhD^a^; Kyung-Il Park, MD, PhD^a, b,c^; Kon Chu, MD, PhD^a^; Ki-Young Jung, MD, PhD^a^; Manho Kim, MD, PhD^a^; Sang Kun Lee, MD, PhD^a^; Jae-Kyu Roh, MD, PhD^d^

^a^ Department of Neurology, Seoul National University Hospital, Seoul, South Korea

^b^ Department of Radiology, Seoul National University Hospital, Seoul, South Korea

^c^ Department of Neurology, Seoul National University Healthcare System Gangnam Center, Seoul, South Korea

^d^ Department of Neurology, The Armed Forces Capital Hospital, Sungnam, South Korea

**List of the supplements: 2 supplemental tables**

**Supplemental Table S1.** Correlation coefficients of sleep/waking ratio of VLF peak power with continuous variables

**Supplemental Table S2.** Univariate analyses for the dichotomized variables with sleep/waking ratio of the VLF peak power

**Supplemental Table S1.** Correlation coefficients of sleep/waking ratio of the VLF peak power with continuous variables

| Ratio of VLF peak power (Sleep/Waking) | r | *P* |
| --- | --- | --- |
| Age (year) | 0.003 | 0.983 |
| Body mass index (kg/m^2^) | 0.183 | 0.203 |
| Systolic blood pressure (mmHg) | -0.105 | 0.469 |
| Diastolic blood pressure (mmHg) | -0.070 | 0.629 |
| Mean blood pressure (mmHg) | -0.105 | 0.466 |
| Average sleep duration (hours) | -0.318 | 0.041^*^ |
| ESS score (0−24) | 0.072 | 0.621 |
| Waking MFV | 0.044 | 0.761 |
| Waking PI | 0.178 | 0.216 |
| MFV variation (Sleep/Waking Ratio) | 0.724 | <0.001^**^ |
| MFV (Sleep/Waking Ratio) | -0.040 | 0.784 |
| PI (Sleep/Waking Ratio) | -0.164 | 0.255 |

ESS, Epworth sleepiness scale, MFV, mean flow velocity, and PI, pulsatility index. ^*^*P*<0.05, ^**^*P*<0.01.

**Supplemental Table S2.** Univariate analyses for the dichotomized variables with sleep/waking ratio of the VLF peak power

|  | sleep/waking ratio of the VLF peak power | | | |
| --- | --- | --- | --- | --- |
|  | No | Yes | *P* |  |
| Male sex | 2.36±2.94 | 3.23±3.15 | 0.414 |  |
| Hypertension | 2.81±3.34 | 3.61±2.40 | 0.420 |  |
| Use of ACEi/ARB | 2.92±3.30 | 3.48±2.21 | 0.616 |  |
| Use of calcium channel blocker | 3.08±3.15 | 2.53±2.82 | 0.733 |  |
| Diabetes mellitus | 2.99±3.25 | 3.44±1.10 | 0.764 |  |
| Hyperlipidemia | 3.07±3.15 | 2.13±1.37 | 0.678 |  |
| Smoking in past 5 years | 3.15±3.71 | 2.87±2.05 | 0.747 |  |
| ESS score >10 | 3.71±1.99 | 2.84±3.34 | 0.415 |  |
| High risk of OSA | 2.93±3.95 | 3.15±1.99 | 0.804 |  |

Data are reported as mean±standard deviation. ACEi/ARB, angiotensin converting enzyme inhibitor/angiotensin receptor blocker, ESS, Epworth sleepiness scale, and OSA, obstructive sleep apnea. ^*^*P*<0.05.
